# Trends and factors contributing to changes in childhood stunting in Bangladesh from 2012 to 2019: A multivariate decomposition modelling

**DOI:** 10.1371/journal.pgph.0004890

**Published:** 2025-07-15

**Authors:** Satyajit Kundu, Richard Gyan Aboagye, Syed Sharaf Ahmed Chowdhury, Md. Hasan Al Banna, Md. Ashfikur Rahman, Rakhi Dey, Faruk Ahmed

**Affiliations:** 1 Faculty of Nutrition and Food Science, Patuakhali Science and Technology University, Patuakhali, Bangladesh; 2 Public Health, School of Medicine and Dentistry, Griffith University, Gold Coast, Queensland, Australia; 3 School of Population Health, Faculty of Medicine and Health, University of New South Wales, Sydney, New South Wales, Australia; 4 Global Health Institute, North South University, Dhaka, Bangladesh; 5 Department of Public Health, North South University, Dhaka, Bangladesh; 6 Development Studies Discipline, Khulna University, Khulna, Bangladesh; 7 Statistics Discipline, Khulna University, Khulna, Bangladesh; PLOS: Public Library of Science, UNITED STATES OF AMERICA

## Abstract

Although the prevalence of childhood stunting has reduced in Bangladesh over time, it is still considered a major public health issue. While research has determined the risk factors for childhood stunting in Bangladesh, the factors that lead to reductions in stunting have received very little attention. Hence, we examined the factors contributing to the changes in childhood stunting over time in Bangladesh using a decomposition approach. In this study, data on childhood stunting of 41,013 under-5 children (U5C) were utilized from the Multiple Indicator Cluster Survey (MICS) 2012 and 2019, which are nation-wide cross-sectional surveys. Mixed-effect logistic regression analysis was adopted to identify the predictors of childhood stunting, and multivariate decomposition analysis was used to examine the factors contributing to the changes in childhood stunting over time. The prevalence of stunting declined from 41.9% in 2012 to 28.0% in 2019. Regression analysis showed that lower education of household head and mothers, older children, lower wealth status of households, unimproved sanitation facilities, and being urban residents were significant predictors of childhood stunting. The decomposition analysis revealed that around 86% of the overall decline in stunting resulted from the differences in the effect of independent variables. Furthermore, children’s age, maternal education, place of residence, and regions were significant factors contributing to the decline in childhood stunting prevalence over time based on both compositional and behavioral changes in these factors. Although childhood stunting has decreased in Bangladesh over time, the current prevalence remains high. Over 86% of the overall decline in stunting was due to the differences in the effect of independent variables. Interventions targeting children of mothers with lower education, infants, rural children, and children from households with lower wealth status and unimproved sanitation facilities may help to reduce the stunting prevalence in Bangladesh.

## Introduction

Malnutrition poses a significant public health concern. It is associated with serious and lasting developmental, economic, social, and medical impacts on individuals and their families, communities, and countries [1,2]. Malnutrition encompasses undernutrition, inadequate vitamins or minerals, overweight/obesity, and diet-related noncommunicable diseases. Malnutrition among children consists of stunting, wasting, and being underweight [1]. In 2022, an estimated 149 million and 45 million children worldwide were stunted and wasted, respectively [1]. Also, nearly half of the under-five mortalities have been linked to undernutrition, with a disproportionate burden among those in low-and middle-income countries [1,3]. Moreover, undernutrition puts children at greater risk of dying from common infections, as well as increases the frequency and severity of those infections and delays the recovery rate [4,5].

Stunting is defined as a low height for age (a child with height-for-age z-score less than − 2 standard deviations) and is a major undernutrition indicator [6]. The United Nations Children's Fund (UNICEF) report indicates that the magnitude of stunting among children under five years has reduced from 204.2 million in 2000 to 148.1 million in 2022 [5]. Also, an estimated two out of five children with stunting live in South Asia, of which Bangladesh is a part, while another two out of five live in sub-Saharan Africa [5]. However, a much faster decline is required to achieve Sustainable Development Goal (SDG) 2, target 2.2, which seeks to reduce and end all forms of malnutrition, including achieving, by 2025, the internationally agreed-upon targets on stunting and wasting in children under 5 years of age [7].

In Bangladesh, stunting remains prevalent among children under five years, with a rate higher than the global rate [8,9]. On average, 31% of children under five in 2017 were stunted according to the Bangladesh Demographic and Health Survey (BDHS) 2017 [10]. A study conducted using data from 2007 to 2017 revealed that stunting among children has reduced from 42% in 2007 to 30% in 2017 [11]. Similarly, the Multiple Indicator Cluster Survey (MICS) 2019 report indicated that stunting has seen a decline from 42% in 2012 to 28% in 2019 [12]. In the same vein, Kumar et al. [13] reported that the prevalence of stunting among children has declined significantly, from 49.8% to 30.7% between two survey periods (2004 and 2017–18). Other studies in Bangladesh have reported the prevalence of stunting among children to vary from 7.97% to 36.3% [14–16]. However, the decline in stunting rate is steady, posing a trend to the country’s ability to achieve the SDG target 2.2 [17].

The risk factors of stunting are multifaceted and multifactorial requiring a holistic approach in dealing with them. According to Stewart et al. [18], the risk factors of stunting encompass household and family factors, inadequate complementary feeding, breastfeeding, infection, and community and societal factors such as water, sanitation, hygiene, education, health and healthcare, and society and culture. Previous studies conducted in Bangladesh have reported that antenatal care, place of residence, parental education, wealth index, religion, child’s age, and mother’s short stature were found to influence stunting in children [8,14,19,20]. Also, other studies have shown the risk factors of stunting to be the sex of the child, low birth weight, body mass index of the mother, household food insecurity, sanitation facilities, divisions, mothers’ involvement in decision-making about children’s healthcare with the father, childhood diarrhoea, and lack of breastfeeding [9,11,13,15,16,19].

Despite several research on childhood stunting in Bangladesh, there remains a significant gap in understanding the specific factors contributing to changes in stunting rates over time using nationally representative data. To address this knowledge gap, our study employs a decomposition analytical method to evaluate the factors influencing changes in stunting prevalence among children under five in Bangladesh from 2012 to 2019. This approach allows for a nuanced understanding of individual factors that either increase or decrease stunting prevalence, providing valuable insights for policymakers and health organizations. Therefore, the objective of this study was to examine the trends and determinants of changes in childhood stunting in Bangladesh during this critical period by utilizing a comprehensive dataset and advanced analytical techniques. Our findings will have significant implications for strengthening existing interventions aimed at reducing stunting prevalence. Moreover, they will inform policymakers in designing and implementing more effective, targeted strategies to prevent and reduce childhood stunting in Bangladesh, particularly focusing on the factors that have a significant influence on the changes in stunting over time.

## Methods

### Data source

This study analyzed two consecutive nationally representative secondary sources of data from the MICS in 2012–13 and 2019 in Bangladesh. The Bangladesh Bureau of Statistics (BBS) and UNICEF collaborated to undertake the MICS in Bangladesh. MICS is considered as the key source of trustworthy statistical evidence on child health globally through a face-to-face interview method directed by skilled field workers that covers a wide range of themes including information on maternal and child health through household surveys [12,21].

### Study design, sampling, and population

**Fig 1:**
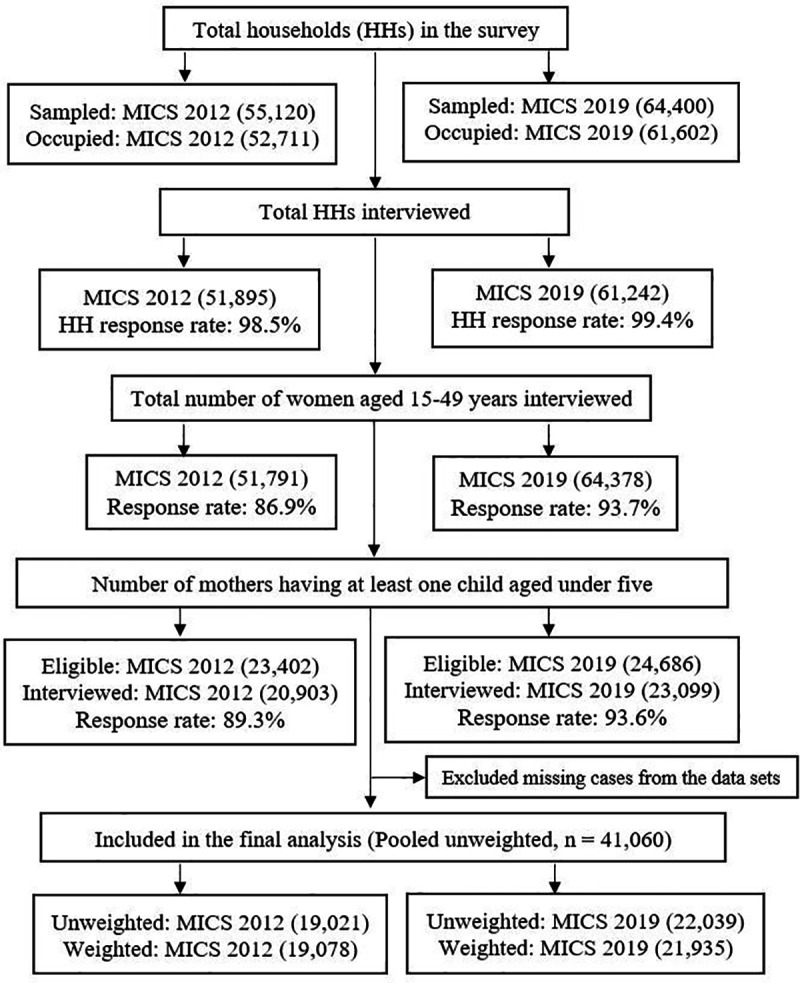
Flow chart of the participants’ selection from MICS 2012 and MICS 2019 data.

A cross-sectional survey was designed at the household (HH) level, where data were collected from 64 districts in Bangladesh. Data from the households were gathered by applying a two-stage stratified cluster sampling technique to guarantee national representation. The enumeration areas (EAs) from the last census in Bangladesh were considered as the primary sampling unit (PSU). A sample of 20 households was taken from each PSU systematically. In the MICS 2012 survey, 51,895 households were interviewed with a total number of eligible women of 51,791, while the MICS 2019 was based on a sample of 61,242 interviewed households with a total number of eligible women of 64,378. Detailed information on the sampling technique, questionnaire, and study procedure can be found elsewhere [12,21]. Children’s data files were used in this study, where a total of 20,903 women in MICS 2012, and 23,099 women in MICS 2019 aged between 15 and 49 years who had at least one child aged under five were interviewed. After excluding all missing cases, the final analysis included data on a total number of 19,078 (weighted) children in MICS 2012, and 21,935 (weighted) children in MICS 2019, yielding a pooled sample of 41,060 children under five. The sample selection and case exclusion from both MICS 2012 and 2019 have been shown in Fig 1.

### Outcome variable

Stunting of under five children was the main outcome variable, which was one of three major anthropometric measures, including height-for-age z (HAZ) scores, weight-for-age z (WAZ) scores, and weight-for-height z (WHZ) scores used for assessing the nutritional status of children [6,22]. Stunting status is calculated using height-for-age z (HAZ) scores according to the World Health Organization’s (WHO) recommended Child Growth Standards [6]. HAZ is a measure of linear growth among the three major anthropometric indices of child growth [8]. MICS calculated HAZ scores using the WHO growth standard as a reference population. Since HAZ scores may both indicate a child’s long-term development and the impacts of chronic malnutrition, the provided z-scores in MICS were used to create the outcome variable. If a child’s height was more than two standard deviations below the reference median for that age group, they were deemed stunted; that is, children with HAZ scores less than − 2 standard deviations were termed stunted [6].

### Explanatory variables

Consistent with previous studies in Bangladesh [8,13,14,23], we included several demographic, household, and child characteristics as explanatory variables in this study. The included explanatory variables are sex and education of household heads, sex and age of children, education of mother, religion, household wealth status, salt iodization test, sanitation facility, floor materials, roof materials, wall materials, place of residence, administrative divisions, number of under-five children, and family size. Education of household head and mothers had four categories (none or pre-primary, primary: 1–5 years, secondary: 6–10 years, and higher: > 10 years) [12]. The age of children was divided into five categories (0 year: 0–11 months, 1 year: 12–23 months, 2 years: 24–35 months, 3 years: 36–47 months, 4 years: 48–59 months). We categorized religion into Muslim and non-Muslim, since more than 90% of the participants in this survey were Muslims [12]. The socioeconomic status of households was determined by using the wealth index. Principal components analysis was used by MICS to generate the wealth index after accounting for the type of housing, type of toilet, type of fuel used for cooking, electricity, bank account, some durable assets, and any animals that may be present in the household. The household wealth index was then divided into five quintiles and categorized as poorest, second, middle, fourth, and richest [12].

A previous study [24] suggested that if the daily minimum intake of iodine is not fulfilled, growth would be impeded, and stunted growth can be prevented. To account for the impact of iodized salt in lowering the risk of stunting in children under five, we consequently added the salt iodization test variable in our analysis. In MICS, each household participated in a salt iodization test, the results of which were divided into four groups: 0 parts per million (PPM) (not iodized), greater than 0 PPM and less than 15 PPM, 15 PPM or more, and no salt in the home. A cut-off point of 15 PPM was used in this survey, meaning that salt with 15 PPM or more of iodate or iodide would be deemed to be sufficiently iodized [12,14]. The source of drinking water and sanitation facilities of a household were categorized into improved and unimproved following the WHO-UNICEF Joint Monitoring Programme for Water Supply, Sanitation and Hygiene (JMP) [25]. Boreholes, piped water or tube wells, protected dug wells, protected springs, tanker trucks, rainwater, and packaged water were all considered improved sources of drinking water; unimproved sources included surface water that was collected directly from rivers, dams, lakes, ponds, streams, canals, and irrigation channels [26]. A flush or pour-flush toilet, a pit latrine with a slab, a vented improved latrine, and a composting toilet were regarded as improved sanitation facilities, but pit latrines without a slab or platform, hanging latrines, or bucket latrines were deemed unimproved sanitation facilities of a household [25]. In this study, floor, roof, and wall materials were categorized into finished and natural materials. The details of the categorization of floor, roof, and wall materials can be found in the MICS report [12] and previous studies [27,28]. We also considered the number of under-five children and the family size of households, keeping these as count variables in our analysis.

### Data analysis

In this study, we used Stata MP version 17.0 (StataCorp, College Station, TX, USA) for data management and statistical analyses. Sample weighting was done using the *‘svy’* command in order to consider the clustering effect and sample stratification in all analyses. Descriptive statistics were estimated for the sample characteristics, and the distribution of prevalence of stunting across sub-groups of all independent variables was shown. After extracting pertinent variables for trends, regression, and multivariate decomposition analysis, the childhood stunting dataset from MICS 2012 and 2019 was appended. The trend period from 2012 to 2019 was assessed to show the change in the weighted prevalence of childhood stunting using descriptive analysis stratified by the selected independent variables. Additionally, a map was generated using ArcGIS (version 10.8) showing the district-wise distribution of change rates of stunting among under-five children in Bangladesh over time. The district-wise change rates of stunting over time were calculated using the following formula:


$$\frac{\left((\%\;of\;stunting\;in\;the\;recent\;year)-(\%\;of\;stunting\;in\;the\;previous\;year)\right)}{\left(\%\;of\;stunting\;in\;the\;previous\;year\right)}\times 100$$

Then, mixed-effect (multilevel) binary logistic regression was employed, keeping the clusters as level-2 factor, since MICS used a two-stage stratified cluster sampling that made the data set a hierarchical composition. Hence, to account for the clustering effect, we applied the multivariable multilevel logistic regression analysis to determine the factors associated with the stunting status of children under five [29]. After applying the multilevel regression model, intraclass correlation (ICC) and Akaike’s information criterion (AIC) were estimated as post-estimation for model fitness. Adjusted odds ratios (AORs) with their 95% confidence intervals (CIs) from the regression analysis were interpreted.

Finally, a non-linear multivariate logit decomposition analysis was done to determine the significant contributing factors to the change in the prevalence of child stunting from 2012 to 2019. In social research, a multivariate decomposition analysis is frequently employed to measure the contributions to group disparities in average predictions using multivariate models. To determine the possible source of the variations in the proportion of children stunting from 2012 to 2019, multivariate decomposition analysis was used. The result of the logistic regression analysis is used by the multivariate decomposition model for the nonlinear response to partition the observed variation in the prevalence of childhood stunting between the surveys into the component parts [30]. Stunting prevalence changes were additionally decomposed into two components: changes in participant composition between surveys (Endowment) and variations in the effect of certain independent variables (Coefficient) [31]. In simpler terms, the endowment component—also called compositional change—captures how differences in characteristics of the population (such as maternal education, wealth status, sanitation facilities, etc) between the two survey years contribute to changes in stunting. The coefficient component—also called behavioral change—reflects how the influence or impact of these characteristics on stunting has shifted over time, which could result from changes in health indicators [30,31].

### Ethics approval

This study did not require any ethical approval as the analysis used only de-identified existing unit record data from the secondary source, MICS.

## Results

### Background characteristics of children

Table 1 shows the distribution of demographic, household, and children’s characteristics in 2012, 2019, and combinedly. A total of 41,013 children’s data (pooled) were analyzed. In these two consecutive surveys, sex and distribution of the age group of children were almost equal. About 35% of household heads and 16% of mothers of children had no formal education or pre-primary education. Around 26% of households did not use iodized salt. Almost 21% of the households’ sanitation facilities were unimproved, while a significant number of households had natural floor (67.7%) and wall (65.0%) materials that were not finished. More than 79% of children were from rural areas in Bangladesh (Table 1).

**Table 1:** Sample characteristics using MICS 2012 and 2019.

Variables	Percentage (weighted) distribution		
**MICS 2012** **(n = 19,078)**	**MICS 2019** **(21,935)**	**Pooled Data** **(n = 41,013)**
**Sex of household head**			
Male	17,904 (93.8)	20,062 (91.5)	37,966 (92.6)
Female	1,174 (6.2)	1,873 (8.5)	3,047 (7.4)
**Education of household head**			
Pre-primary or none	7,633 (40.0)	6,590 (30.0)	14,223 (34.7)
Primary	5,150 (27.0)	6,467 (29.5)	11,617 (28.3)
Secondary	3,554 (18.6)	6,128 (27.9)	9,682 (23.6)
Higher+	2,741 (14.4)	2,750 (12.6)	5,491 (13.4)
**Sex of the child**			
Male	9,750 (51.1)	11,392 (51.9)	21,142 (51.6)
Female	9,328 (48.9)	10,543 (48.1)	19,871 (48.4)
**Age of the child**			
0 year	3,658 (19.2)	4,377 (20.0)	8,034 (19.6)
1 year	3,817 (20.0)	4,264 (19.4)	8,081 (19.7)
2 years	3,769 (19.7)	4,300 (19.6)	8,070 (19.7)
3 years	3,925 (20.6)	4,578 (20.9)	8,503 (20.7)
4 years	3,909 (20.5)	4,416 (20.1)	8,325 (20.3)
**Education of mother**			
Pre-primary or none	4,229 (22.1)	2,462 (11.2)	6,690 (16.3)
Primary	5,658 (29.7)	5,212 (23.8)	10,870 (26.5)
Secondary	6,710 (35.2)	10,778 (49.1)	17,489 (42.7)
Higher+	2,481 (13.0)	3,483 (15.9)	5,964 (14.5)
**Religion**			
Muslim	17,422 (91.3)	20,080 (91.5)	37,502 (91.4)
Non-Muslim	1,656 (8.7)	1,855 (8.5)	3,511 (8.6)
**Household wealth status**			
Poorest	4,608 (24.2)	4,766 (21.7)	9,375 (22.9)
Second	3,896 (20.4)	4,333 (19.7)	8,228 (20.0)
Middle	3,539 (18.6)	4,120 (18.8)	7,658 (18.7)
Fourth	3,461 (18.1)	4,294 (19.6)	7,755 (18.9)
Richest	3,574 (18.7)	4,422 (20.2)	7,996 (19.5)
**Salt iodization test**			
0 ppm (not iodized)	5,125 (26.9)	5,316 (24.2)	10,441 (25.5)
<15 ppm	3,462 (18.1)	3,540 (16.1)	7,001 (17.1)
≥ 15 ppm	10,253 (53.7)	12,975 (59.2)	23,229 (56.6)
No salt in the household	238 (1.3)	104 (0.5)	342 (0.8)
**Source of drinking water**			
Improved	18,750 (98.3)	21,591 (98.4)	40,341 (98.4)
Unimproved	328 (1.7)	344 (1.6)	672 (1.6)
**Sanitation facility**			
Improved	14,185 (74.4)	18,258 (83.2)	32,442 (79.1)
Unimproved	4,893 (25.6)	3,677 (16.8)	8,571 (20.9)
**Floor material**			
Finished	4,765 (25.0)	8,498 (38.7)	13,264 (32.3)
Natural	14,313 (75.0)	13,437 (61.3)	27,749 (67.7)
**Roof material**			
Finished	18,486 (96.9)	21,728 (99.1)	40,214 (98.1)
Natural	592 (3.1)	207 (0.9)	799 (1.9)
**Wall material**			
Finished	6,103 (32.0)	8,272 (37.7)	14,375 (35.0)
Natural	12,975 (68.0)	13,663 (62.3)	26,638 (65.0)
**Place of residence**			
Urban	3,931 (20.6)	4,569 (20.8)	8,499 (20.7)
Rural	15,147 (79.4)	17,366 (79.2)	32,514 (79.3)
**Administrative division**			
Barishal	1,143 (6.0)	1,226 (5.6)	2,369 (5.8)
Chattogram	4,218 (22.1)	4,712 (21.5)	8,931 (21.8)
Dhaka	6,037 (31.6)	6,864 (31.3)	12,901 (31.5)
Khulna	1,892 (9.9)	2,282 (10.4)	4,174 (10.2)
Rajshahi	2,234 (11.7)	2,662 (12.1)	4,896 (11.9)
Rangpur	2,227 (11.7)	2,415 (11.0)	4,642 (11.3)
Sylhet	1,327 (7.0)	1,774 (8.1)	3,100 (7.5)
**Number of under 5 children**			
Median (IQR)	1 (1 – 2)	1 (1 – 2)	1 (1 – 2)
Mean (SD)	1.3 (0.6)	1.3 (0.5)	1.3 (0.6)
**Number of family members**			
Median (IQR)	5 (4 – 6)	5 (4 – 6)	5 (4 – 6)
Mean (SD)	5.6 (2.3)	5.4 (2.2)	5.5 (2.2)

ppm: parts per million; IQR: Inter Quartile Range; SD: Standard Deviation;

### Trends in the prevalence of stunting status of children

The trend of prevalence of stunting among U5C over time (2012–2019) indicates a significant decrease from 41.9% in 2012 to 28.0% in 2019. The percent point differences in stunting prevalence also show a significant decline across sub-groups of each variable. For example, a 14.9%-point reduction was observed in rural areas, compared to a 10.1%-point reduction in urban areas. Considering the wealth status of households, the highest decrease was identified among the middle wealth group (16.2%-point). Among the administrative divisions in Bangladesh, the highest prevalence of stunting among U5C was observed in Sylhet in both 2012 and 2019, with a decline of 13.5%-point from 2012 to 2019. However, the highest decline of stunting prevalence was found in the Rangpur division (17.0%-point) (Table 2).

**Table 2:** Trends of prevalence of childhood stunting in Bangladesh, segregated by survey year, MICS 2012 and 2019.

Variables	Prevalence (weighted) of stunting (%)			Difference in point estimates
**Pooled Data**	**MICS 2012**	**MICS 2019**	**2012 – 2019**
**Overall**	34.4	41.9	28.0	13.9
**Sex of household head**				
Male	34.7	42.0	28.1	13.9
Female	31.6	40.0	26.3	13.7
**Education of household head**				
Pre-primary or none	40.8	47.6	32.9	14.7
Primary	37.1	45.4	30.4	15.0
Secondary	28.6	36.0	24.4	11.6
Higher+	22.7	27.2	18.4	8.8
**Sex of the child**				
Male	34.6	42.3	28.0	14.3
Female	34.3	41.5	27.9	13.6
**Age of the child**				
0 year	21.4	24.9	18.4	6.5
1 year	36.0	42.4	30.4	12.0
2 years	41.6	49.2	35.0	14.2
3 years	39.8	50.0	31.1	18.9
4 years	33.1	42.1	25.1	17.0
**Education of mother**				
Pre-primary or none	47.2	51.4	40.0	11.4
Primary	42.1	49.5	34.2	15.3
Secondary	29.5	36.4	25.2	11.2
Higher+	20.6	23.1	18.7	4.4
**Religion**				
Muslim	34.5	42.1	27.9	14.2
Non-Muslim	34.2	39.9	29.2	10.7
**Household wealth status**				
Poorest	45.3	52.8	38.1	14.7
Second	38.8	46.9	31.5	15.4
Middle	33.5	42.2	26.0	16.2
Fourth	29.5	36.9	23.5	13.4
Richest	23.0	26.9	19.8	7.1
**Salt iodization test**				
0 ppm (not iodized)	39.5	48.0	31.2	16.8
<15 ppm	38.6	45.9	31.4	14.5
≥ 15 ppm	30.9	37.5	25.7	11.8
No salt in the household	37.2	38.2	35.0	3.2
**Source of drinking water**				
Improved	34.3	41.8	27.9	13.9
Unimproved	40.5	47.7	33.5	14.2
**Sanitation facility**				
Improved	31.8	38.	26.5	11.5
Unimproved	44.6	51.6	35.2	16.4
**Floor material**				
Finished	25.6	31.4	22.4	9.0
Natural	38.7	45.4	31.5	13.9
**Roof material**				0.0
Finished	34.1	41.5	27.9	13.6
Natural	50.1	53.8	39.2	14.6
**Wall material**				
Finished	27.1	33.5	22.4	11.1
Natural	38.4	45.8	31.3	14.5
**Place of residence**				
Urban	30.9	36.3	26.2	10.1
Rural	35.4	43.3	28.4	14.9
**Administrative division**				
Barishal	36.1	42.1	30.5	11.6
Chattogram	34.5	42.8	27.1	15.7
Dhaka	35.3	42.0	29.3	12.7
Khulna	26.7	34.2	20.5	13.7
Rajshahi	32.3	39.3	26.4	12.9
Rangpur	34.7	43.6	26.6	17.0
Sylhet	43.0	50.8	37.3	13.5

ppm: parts per million.

**Fig 2:**
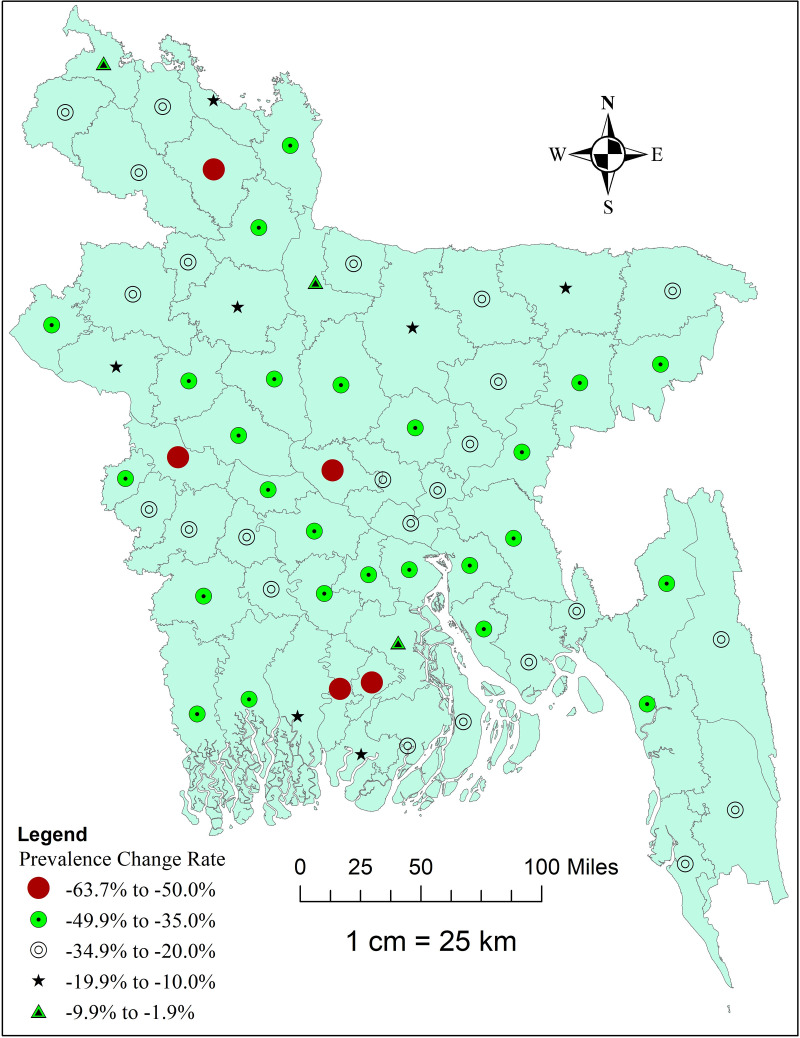
Map showing the district-wise change rate in the prevalence of stunting among U5C in Bangladesh, MICS 2012 to 2019. This map was generated using data from tource: https://data.humdata.org/dataset/cod-ab-bgd?.

Fig 2 illustrates the district-wise change rate in the prevalence of stunting among U5C in Bangladesh from 2012 to 2019. The highest rate of decline was observed in two southern districts (Pirojpur and Jhalokathi), a western-side district (Kushtia), and a northern district (Rangpur), while the lowest decline was found in the most northern side district (Panchagarh) and Jamalpur district (Fig 2).

### Factors associated with stunting status

#### Measures of variation (random-effects).

The intercept-only regression models (null model) indicated that the likelihood of children from various clusters having stunting status varied significantly (in pooled data, variance: 0.21, 95% CI: 0.18-0.24), indicating the significance of applying multilevel modelling. The intraclass correlation value of the null model suggested that 8% in 2012 and 10% in 2019 of the total variation in childhood stunting was due to the differences from cluster to cluster, while the variation was 6% for the pooled data (Table 3).

**Table 3:** Multilevel regression analysis of risk factors for stunting among children under 5 in Bangladesh, MICS 2012 and 2019.

Variables	MICS 2012	MICS 2019	Pooled Data^#^
***Null model (Intercept only)***			
Cluster level variance (95% CI)	0.29 (0.24, 0.34)	0.38 (0.32, 0.44)	0.21 (0.18, 0.24)
Log-likelihood	-12794.1	-12805.1	-26151.7
Intraclass correlation (SE)	0.08 (0.006)	0.10 (0.007)	0.06 (0.004)
Akaike’s information criterion	25592.2	25614.1	52307.5
***Adjusted model***	**AOR (95% CI)**	**AOR (95% CI)**	**AOR (95% CI)**
**Sex of household head**			
Male	0.95 (0.83, 1.10)	0.99 (0.88, 1.12)	0.98 (0.90, 1.07)
Female (Ref)	1.00	1.00	1.00
**Education of household head**			
Pre-primary or none	1.20** (1.05, 1.36)	1.23** (1.07, 1.42)	1.22*** (1.12, 1.34)
Primary	1.25** (1.10, 1.43)	1.26** (1.10, 1.44)	1.26*** (1.15, 1.37)
Secondary	1.04 (0.91, 1.18)	1.15* (1.00, 1.31)	1.10* (1.01, 1.21)
Higher+ (Ref)	1.00	1.00	1.00
**Sex of the child**			
Male	1.04 (0.97, 1.10)	1.03 (0.96, 1.09)	1.03 (0.98, 1.08)
Female (Ref)	1.00	1.00	1.00
**Age of the child**			
0 year (Ref)	1.00	1.00	1.00
1 year	2.24*** (2.18, 2.69)	2.03*** (1.83, 2.27)	2.19*** (2.04, 2.36)
2 years	3.19*** (2.87, 3.55)	2.57*** (2.31, 2.86)	2.81*** (2.61, 3.02)
3 years	3.30*** (2.97, 3.67)	2.10*** (1.89, 2.34)	2.58*** (2.40, 2.78)
4 years	2.24*** (2.02, 2.49)	1.47*** (1.32, 1.64)	1.81*** (1.67, 1.94)
**Education of mother**			
Pre-primary or none	1.79*** (1.54, 2.08)	1.69*** (1.45, 1.96)	1.70*** (1.53, 1.88)
Primary	1.95*** (1.69, 2.24)	1.43*** (1.26, 1.63)	1.67*** (1.53, 1.83)
Secondary	1.42*** (1.25, 1.62)	1.14* (1.02, 1.28)	1.26*** (1.16, 1.37)
Higher+ (Ref)	1.00	1.00	1.00
**Religion**			
Muslim (Ref)	1.00	1.00	1.00
Non-Muslim	0.99 (0.87, 1.12)	1.04 (0.91, 1.18)	1.00 (0.92, 1.08)
**Household wealth status**			
Poorest	2.20*** (1.81, 2.68)	2.59*** (2.13, 3.15)	2.24*** (1.96, 2.55)
Second	2.00*** (1.69, 2.34)	2.14*** (1.77, 2.58)	1.94*** (1.71, 2.20)
Middle	1.77*** (1.48, 2.12)	1.70*** (1.44, 2.01)	1.64*** (1.46, 1.84)
Fourth	1.41*** (1.22, 1.63)	1.39*** (1.22, 1.59)	1.36*** (1.24, 1.49)
Richest (Ref)	1.00	1.00	1.00
**Salt iodization test**			
0 ppm (not iodized) (Ref)	1.00	1.00	1.00
<15 ppm	0.99 (0.90, 1.10)	1.04 (0.94, 1.15)	1.01 (0.94, 1.08)
≥ 15 ppm	0.93 (0.85, 1.01)	0.92 (0.85,1.01)	0.92** (0.86, 0.98)
No salt in the household	0.72* (0.54, 0.97)	1.39 (0.89, 2.18)	0.88 (0.69, 1.12)
**Source of drinking water**			
Improved (Ref)	1.00	1.00	1.00
Unimproved	0.87 (0.67, 1.13)	0.91 (0.69, 1.20)	0.90 (0.75, 1.09)
**Sanitation facility**			
Improved (Ref)	1.00	1.00	1.00
Unimproved	1.22*** (1.12, 1.32)	1.03 (0.94, 1.13)	1.13*** (1.06, 1.19)
**Floor material**			
Finished (Ref)	1.00	1.00	1.00
Natural	0.89 (0.77, 1.02)	0.96 (0.85, 1.08)	0.94 (0.87, 1.03)
**Roof material**			
Finished (Ref)	1.00	1.00	1.00
Natural	1.02 (0.84, 1.25)	1.24 (0.90, 1.72)	1.06 (0.90, 1.25)
**Wall material**			
Finished (Ref)	1.00	1.00	1.00
Natural	1.05 (0.95, 1.16)	0.93 (0.84, 1.03)	0.99 (0.92, 1.06)
**Place of residence**			
Urban	1.01 (0.91, 1.12)	1.26*** (1.13, 1.41)	1.14*** (1.06, 1.22)
Rural (Ref)	1.00	1.00	1.00
**Administrative division**			
Barishal	0.85 (0.73, 1.00)	0.88 (0.75, 1.04)	0.86* (0.77, 0.97)
Chattogram	1.06 (0.95, 1.19)	0.92 (0.82, 1.03)	0.96 (0.89, 1.04)
Dhaka (Ref)	1.00	1.00	1.00
Khulna	0.73*** (0.63, 0.84)	0.63*** (0.55, 0.73)	0.68*** (0.62, 0.75)
Rajshahi	0.82** (0.72, 0.94)	0.80** (0.70, 0.91)	0.79*** (0.73, 0.86)
Rangpur	0.94 (0.83, 1.07)	0.77*** (0.67, 0.88)	0.85*** (0.78, 0.92)
Sylhet	1.33*** (1.13, 1.55)	1.37*** (1.17, 1.60)	1.31*** (1.18, 1.45)
**Number of under 5 children**	1.10** (1.03, 1.18)	1.17*** (1.09, 1.25)	1.13*** (1.08, 1.19)
**Number of family members**	1.00 (0.98, 1.02)	0.98 (0.96, 1.00)	0.99 (0.98, 1.00)
**Variance and model fitness**			
Cluster level variance (95% CI)	0.22 (0.17, 0.26)	0.31 (0.26, 0.37)	0.15 (0.13, 0.18)
Log-likelihood	-12006.8	-12257.4	-24417.7
Intraclass correlation (SE)	0.06 (0.006)	0.09 (0.007)	0.04 (0.004)
Akaike’s information criterion	24085.6	24586.9	48909.4
Significance level of model^$^	p < 0.001	p < 0.001	p < 0.001

# Survey year was adjusted for pooled regression. AOR: Adjusted Odds Ratio; CI: Confidence Interval; ppm: parts per million; SE: Standard Error. ^$^ Significance of cluster-level random effects was assessed using likelihood ratio tests (comparing models with and without random effects).

* P < 0.05, ** P < 0.01, *** P < 0.001.

#### Measures of associations (fixed-effects).

The multilevel regression analysis estimated the significant factors associated with stunting status among U5C in Bangladesh. Our findings revealed that the educational level of both household heads and mothers significantly influenced childhood stunting. Children from households having household heads or mothers with lower education faced higher odds of stunting compared to those from families with higher educational attainment. Similarly, older children were more likely to be stunted compared to younger children. For example, children aged 4 years (48–59 months) had an 81% higher likelihood of being stunted compared to those aged 0–11 months (AOR: 1.81, 95% CI: 1.67-1.94). Children from poorer households were comparatively more stunted than those from wealthy households. For instance, children from households with the poorest wealth index were 2.24 times higher likely to be stunted compared to those from the richest households (AOR: 2.24, 95% CI: 1.96-2.55). When household members consumed iodized salt with ≥ 15 ppm, children of those households showed lower odds of being stunted compared to those from households with no iodized salt (AOR: 0.92, 95% CI: 0.86-0.98). When looking at the sanitation facilities, children from households having unimproved sanitation facilities were more likely to be stunted compared to those from households with improved sanitation facilities (AOR: 1.13, 95% CI: 1.06-1.19). In this study, we found a rural-urban difference, while urban children were more stunted than rural children (AOR: 1.14, 95% CI: 1.06-1.22); however, this finding was not significant in 2012 (Table 3).

### Findings of multivariate decomposition analysis

The trend analysis showed an overall decline in the prevalence of stunting among U5C in Bangladesh. The decomposition analysis determined that a decline in the prevalence of stunting over time was explained by the differences in the included explanatory variables and the effects of the characteristics over time, 2012–2019 (Table 4).

**Table 4:** Multivariate decomposition analysis of change in childhood stunting in Bangladesh from 2012 to 2019.

Variables	Difference due to characteristics (E)		Difference due to coefficient (C)	
**Coefficient**	**Percent**	**Coefficient**	**Percent**
Overall decomposition of the change	-0.01923***	13.8	-0.11987***	86.2
Overall residual (SE)	-0.13910*** (0.00451)			
**Sex of household head**				
Male	0.00005	-0.04	0.00595	-4.28
Female (Ref)				
**Education of household head**				
Pre-primary or none	-0.00451**	3.24	0.00360	-2.59
Primary	0.00114**	-0.82	0.00015	-0.11
Secondary	0.00238	-1.71	0.00334	-2.40
Higher+ (Ref)				
**Sex of the child**				
Male	0.00003	-0.02	-0.00202	1.45
Female (Ref)				
**Age of the child**				
0 year (Ref)				
1 year	-0.00081***	0.59	-0.00784*	5.63
2 year	-0.00029**	0.21	-0.00914**	6.57
3 year	0.00044***	-0.32	-0.01944***	13.97
4 year	-0.00028***	0.20	-0.01839***	13.22
**Education of mother**				
Pre-primary or none	-0.01183***	8.50	-0.00616	4.43
Primary	-0.00447***	3.22	-0.02271***	16.33
Secondary	0.00400*	-2.88	-0.01937**	13.92
Higher+ (Ref)				
**Religion**				
Muslim (Ref)				
Non-Muslim	-0.00002	0.01	0.00199	-1.43
**Household wealth status**				
Poorest	-0.00435***	3.13	0.00740	-5.32
Second	-0.00093***	0.67	0.00129	-0.93
Middle	0.00022***	-0.15	-0.00323	2.32
Fourth	0.00084***	-0.60	-0.00224	1.61
Richest (Ref)				
**Salt iodization test**				
0 ppm (not iodized) (Ref)				
<15 ppm	-0.00010	0.07	0.00075	-0.54
≥ 15 ppm	-0.00089	0.64	-0.00056	0.41
No salt in the household	-0.00046	0.33	0.00151*	-1.08
**Source of drinking water**				
Improved (Ref)				
Unimproved	0.00005	-0.04	-0.00020	0.14
**Sanitation facility**				
Improved (Ref)				
Unimproved	-0.00085	0.61	-0.00882**	6.34
**Floor material**				
Finished (Ref)				
Natural	0.00081	-0.58	0.02191	-15.75
**Roof material**				
Finished (Ref)				
Natural	-0.00047	0.34	0.00056	-0.40
**Wall material**				
Finished (Ref)				
Natural	0.00088	-0.63	-0.02089*	15.02
**Place of residence**				
Urban	0.00011***	-0.08	0.00873**	-6.28
Rural (Ref)				
**Administrative division**				
Barishal	0.00012	-0.09	0.00048	-0.34
Chattogram	0.00015*	-0.11	-0.00695*	5.00
Dhaka (Ref)	1.00			
Khulna	0.00048***	0.35	-0.00350	2.51
Rajshahi	-0.00022***	0.15	-0.00097	0.70
Rangpur	0.00042***	-0.30	-0.00600**	4.31
Sylhet	0.00060***	-0.43	0.00020	-0.14
**Number of under 5 children**	-0.00117***	0.84	0.01516	-10.90
**Number of family members**	0.00067	-0.48	-0.01163	8.36

ppm: parts per million; SE: Standard Error. * P < 0.05, ** P < 0.01, *** P < 0.001.

#### Difference due to characteristics (Endowment).

The multivariate decomposition analysis revealed that about 14% of the overall decline in stunting was due to the difference in the composition of included variables between the two survey waves. Education of household heads and mothers, age of the children, household wealth status, place of residence, and administrative division were significantly responsible for the change in the prevalence of stunting over time. A decrease in the number of household heads (3.2%) and mothers (8.5%) having pre-primary/no formal education had a positive contribution to the decline of stunting. Similarly, a decrease in the composition of children from the poorest households showed a positive effect on the decline of stunting by 3.1%. An increase in the composition of children from urban areas showed an inverse contribution to the change in stunting (Table 4).

#### Difference due to effects of coefficient.

The decomposition analysis revealed that around 86% of the overall decline in stunting resulted from the behavioural change (differences in the effect of independent variables) towards the stunting status among U5C. Controlling for the roles of compositional change, factors including the age of children, education of mother, iodization of salt, sanitation facility, wall material, and place of residence had significant effects on the coefficient contribution to the estimated change in stunting prevalence over time. Compared to children aged 0–11 months (0 years), the behavioral change in children aged 3, and 4 years contributed 13.9% and 13.2%, respectively, to the decline of stunting over time. The change in the behavior of mothers having primary, and secondary education contributed 16.3% and 13.9% to the observed change in stunting prevalence. Again 6.3% of the decline of stunting over time resulted from the behavioral change of children from households with unimproved sanitation facilities. Similarly, 15% of decrement in stunting over time was observed due to the behavioral change of children from households with natural wall materials. On the contrary, children from urban areas (6.3%), and the number of U5C in the household (10.9%) showed a reversal effect on the decline of stunting prevalence. Furthermore, behavioral change of children from Chattogram (5.0%) and Rangpur (4.3%) divisions positively contributed to the decline of stunting over time (Table 4).

## Discussion

Over the last two decades, Bangladesh has been following a similar global trend in substantial improvement of the undernutrition status, while there is a subsequent increase in overweight and obesity [32,33]. During this period, Bangladesh’s government has implemented different policies and interventions at both the governmental and non-governmental levels [34] to end all forms of undernutrition among children by 2030, aligning with the targets and goals of the SDGs [35]. Evidence suggests that the risk factors for malnutrition have also changed over time [32,36]. In this study, we examined the factors contributing to the changes in childhood stunting over time in Bangladesh using a decomposition approach. We found that stunting prevalence decreased from 41.9% in 2012 to 28.0% in 2019. The significant predictors of childhood stunting include older children (12–59 months), poorer family financial status, inadequate sanitation facilities, living in an urban area, lower education levels of mothers and household heads. The decomposition analysis showed that children’s age, mother education, place of residence, and regions were major factors contributing to the drop in the prevalence of childhood stunting over time based on both compositional and behavioral changes in these characteristics.

Compared to the previous decomposition analysis models, multivariate decomposition logistic regression analysis relaxes the non-linear models [30]. This model was used to examine the trend of stunting and identify the factors contributing positively or negatively to the change in stunting status in children from 2012 to 2019. This study observed the behavioral (coefficient) change of the variables that contributed more in terms of reducing stunting among U5C compared to the compositional (endowment) changes of the variables. A significant downward trend was observed in the stunting level in U5C over the last decade, mainly between the years of 2012–2019.

The age of children was found to be significantly associated with the stunting status. With the increase in the age of children, the odds of stunting were found to increase in the pooled analysis. On decomposition analysis, the age of children was found to be a negative contributor to reducing stunting over time. This finding was found in line with the findings of the previous study conducted in Bangladesh [37,38]. This finding could be possibly explained by the decreased amount of breastfeeding among the children with the increase of age beyond infancy. Breastfeeding was found to be a protective factor against malnutrition [39–41]. Besides, with the increase in the age of children, they are supposed to start with complementary feeding, the improper and inadequate use of which can make the children vulnerable to malnutrition [42] resulting in conditions like stunting.

The lower educational level of the mother was found to be associated with the higher odds of stunting among U5C. The lower educational level of the mother was also found to negatively contribute to reducing stunting over the last decade. This finding coincides with the findings in a previous study [43]. This finding could be attributed to the role of women in ensuring food security for their children and taking care of their children daily in developing countries. A child’s nutritional status is heavily dependent on these two factors [44]. Besides, in a low-resource setting country like Bangladesh, educated women can use the limited family resources to promote better health-related behavior, and by limiting family size [45,46], which may contribute to the decline of stunting in U5C.

In this study, the regression analysis showed that children from households with unimproved sanitation facilities had higher odds of stunting status in U5C. While on decomposition analysis, the unimproved sanitation facility of the household was found to contribute negatively to reducing stunting in children over time. This finding is similar to the findings in previous studies [47–49]. The linear synergistic relationship found between sanitation facilities and childhood stunting could be a probable explanation behind this finding [50–52]. Additionally, the feco-oral route is the main source of transmission of bacteria in children, which could also be a possible link behind this finding.

Stunting was shown to be highly correlated with the wealth status of the household; the likelihood of having a child with stunting increased with a lower wealth status. It also contributed significantly to reducing stunting over the years. A similar finding was observed in the studies conducted in Bangladesh [38,53–55], Congo [56], Ethiopia [57], and other countries [58] previously. This finding could be plausibly explained by the obvious better living conditions of the wealthier families with better household arrangements, including better sanitation facilities and higher access to healthcare facilities [59–63]. Besides, wealthier families can usually spend more money to buy healthier food for their children [64]. Also, rich families distribute their resources comprehensively to their children, meeting the nutritional demands of all children [55]. The apparent difference in resource allocation, housing and health facilities, and food security could be the credible explanation behind this finding, since socioeconomic deprivation is found to be correlated with higher prevalence of malnutrition [64].

Higher odds of stunting were observed among the children living in urban areas compared to those from rural areas. The difference in residence was also found to be a significant contributor to reducing stunting in children in Bangladesh. A previous study among Bangladeshi children revealed similar results [55]. The more likely explanation behind this finding could be that urban parents are mostly educated and working. The maid remains in charge of the kids’ feeding and nutrition for most of the day, which could make them vulnerable to malnutrition, resulting in stunting [55].

Among the administrative divisions, children in Sylhet were found to have higher odds of being stunted than the children in the Dhaka division. In a previous Bangladeshi study similar result was observed [55]. Several factors may underlie this persistent burden. The probable reason behind this finding could be due to the sociocultural disparities among the administrative divisions [55]. Besides, the lower educational rate in the Sylhet division could be another possible explanation behind this finding [65]. Studies have also pointed to lower utilization of maternal and child health services and poor antenatal care coverage in the region [66]. Additionally, household food insecurity has been reported to be more prevalent in Sylhet, further exacerbating the risk of undernutrition among children, especially those from poor and very poor households [67]. These findings suggest the need for region-specific programmatic responses to address the barriers contributing to stunting reduction in Sylhet.

### Policy recommendations

Based on the findings of our study, several policy recommendations can be made to further reduce the prevalence of childhood stunting in Bangladesh. Firstly, policymakers could focus on enhancing educational opportunities for women and household heads should be prioritized, as higher maternal and household head education levels are associated with reduced stunting rates. Implementing community-based educational programs and providing incentives for school attendance could significantly contribute to this goal. Secondly, addressing socioeconomic disparities is crucial; policies aimed at improving the economic conditions of lower-income households, such as microfinance initiatives or targeted subsidies, could alleviate the impact of poverty on child nutrition. Thirdly, improving access to sanitation facilities is essential for reducing stunting. Investment in infrastructure to provide clean water and improved sanitation would help reduce the stunting prevalence. Lastly, targeted interventions in urban areas, where stunting remains a significant issue, should focus on enhancing healthcare access and nutritional support for vulnerable populations. By implementing these strategies, policymakers can address the multifaceted determinants of stunting and promote better health outcomes for children across Bangladesh.

### Strengths and Limitations

The use of nationally representative data makes the study findings generalizable. Besides, the analytical technique included multilevel modeling in regression analysis, considering the hierarchical structure of sampling to consider the cluster effects. Also, the non-linear multivariate decomposition approach to identify the factors influencing the decline of stunting makes the result more robust. However, the study has some limitations too. The cross-sectional nature of the survey limits us to draw any causal inference among the contributing factors and stunting status. We could not consider the factors related to the divisional disparities in the prevalence of stunting that may have affected the association and contribution of the administrative division with stunting. In addition, the analysis was limited by the unavailability of certain important individual-level variables in the MICS datasets, such as comprehensive dietary assessment, utilization of health services, and different cultural factors. The absence of these factors may have introduced omitted variable bias, possibly affecting the accuracy of the estimated effects and limiting a more comprehensive understanding of the determinants of childhood stunting reduction.

## Conclusion

In Bangladesh, stunting is highly prevalent, which is one of the preventable public health issues. The study revealed the significant association of several maternal, child, and geographic variables with stunting and their positive and negative contribution to reducing the burden of stunting over time. The direction of contribution by the factors in reducing the stunting observed from this study will help the policymaker to focus on the specific factors and make a focused policy addressing the negative contributors to improve the stunting status of U5C in the country to eventually meet the goals of SDG. In addition, addressing the urban-rural, and regional disparities through a targeted comprehensive program and intervention targeting elderly children, less wealthy households, and lower-educated mothers could effectively reduce the prevalence of stunting in Bangladesh. This urges the further need for longitudinal studies to find the causal association of these variables with stunting.
